# Multiplex Tests for Respiratory Tract Infections: The Direct Utility of the FilmArray Respiratory Panel in Emergency Department

**DOI:** 10.1155/2020/6014563

**Published:** 2020-07-25

**Authors:** Siyuan Yang, Hui Li, Yunxia Tang, Fengting Yu, Chengjie Ma, Huimin Zhang, Lin Pang, Hongxin Zhao, Linghang Wang

**Affiliations:** ^1^Laboratory of Infectious Diseases Center of Beijing Ditan Hospital, Capital Medical University, Beijing 10015, China; ^2^Emergency Department of Infectious Diseases of Beijing Ditan Hospital, Capital Medical University, Beijing 10015, China; ^3^Department of Pediatrics of Beijing Ditan Hospital, Capital Medical University, Beijing 10015, China; ^4^Clinical and Research Center of Infectious Diseases of Beijing Ditan Hospital, Capital Medical University, Beijing 10015, China; ^5^Institute of Infectious Diseases, Beijing Ditan Hospital, Capital Medical University, Beijing Key Laboratory of Emerging Infectious Diseases, Beijing 100015, China

## Abstract

**Background:**

The FilmArray Respiratory Panel with multiplex targets for respiratory pathogens has been widely used and verified in clinical trials in special test settings. However, it is necessary to evaluate the panel's performance at the point of care directly, in nonspecific test settings.

**Methods:**

Patients with respiratory tract infections were enrolled from among emergency department visitors, and all steps, including the collection of specimens and testing, were performed by our clinicians.

**Results:**

Among 270 patients, 196 (72.6%) patients were found to have one or more pathogens. For signal pathogen detection, influenza A virus had the highest rate of detection; 45 (16.7%) of the patients had two or more respiratory pathogens codetected, and most of the multiplex pathogens were rhinovirus/enterovirus codetected with *Bordetella pertussis* (17.8%). The information provided by the FilmArray had an impact on the prescription of antimicrobials, and there were differences in the rates of antibiotic prescriptions and anti-influenza prescriptions among patients.

**Conclusions:**

Use of the FilmArray by clinical staff was successfully implemented in the emergency department for the first time in China. The FilmArray has the potential for point-of-care testing in nonspecific settings.

## 1. Introduction

Respiratory tract infections (RTIs) are a significant cause of morbidity and mortality worldwide, particularly during the winter months due to seasonal respiratory epidemics [1–4]. In addition to the morbidity and mortality associated with RTIs, the infections also represent a huge impact on emergency department (ED) visits, outpatient medical visits, hospital admissions, and the burden of antimicrobial prescriptions, especially in developing countries because of the absence of rapid and accurate laboratory-dependent testing for respiratory pathogens [5–7]. The majority of RTIs are caused by respiratory viruses, followed by infections of bacterial pathogens [8, 9]. A principal challenge for clinical staff is to distinguish among RTIs with similar symptoms and clinical signs, such as discriminating influenza from the “common cold” or bacterial community-acquired pneumonia (CAP) [10]. Empiric treatment with antibiotics is frequently initiated in patients despite the likelihood of viral infection being the etiology of a patient's symptoms, the potential challenge of bacterial resistance, and even environmental pollution [11, 12]. Definitive etiological diagnosis based on rapid testing and the ability to distinguish multiple respiratory pathogens may improve this situation.

Compared to traditional tests such as direct fluorescence assays (DFA) and cell cultures, laboratory-developed polymerase chain reaction (PCR) tests have high sensitivity and specificity for the detection of respiratory pathogens, with shorter turnaround times (TAT) [13]. The FilmArray Respiratory Panel (BioFire Diagnostics; Salt Lake City, UT, USA) is an automated multiplex molecular system for the detection of 17 viruses, 2 atypical pathogens, and a bacterium in 65 min. Improved influenza detection and antimicrobial prescriptions and reduced length of stay for this system have been widely proven by many clinical trials [1, 14–18]. As an automated multipathogen detection system, the FilmArray RP is an integrated diagnostic system which is rapid, accurate, and reproducible, and in addition, the system minimizes the need for professional laboratory facilities and trained technicians [19]. Though defined as a multipathogen molecular detection of point-of-care (POC) test, the operators of this system, from pouch preparation to data analysis, tend to be experienced laboratory staff who have participated in previously published articles, and general laboratories or microbiology laboratories in hospitals are the only places currently using this multipathogen test. Thus, understanding the direct utility of the FilmArray RP as a POC test for use with outpatients is important.

In this study, we undertook to describe the direct utility of the FilmArray RP as a POC test in our emergency department, which is known to manage a significant number of infectious diseases during the winter months due to seasonal respiratory epidemics among ED visitors. The aim of this study was to investigate the performance of the FilmArray RP system when utilized in a nonspecific test setting and with clinicians in a hands-on manner.

## 2. Materials and Methods

### 2.1. Study Design and Participants

This single-center, open, observational study took place between October 2017 and February 2018 in Beijing Ditan Hospital (BJDH), affiliated with Capital Medical University, China. BJDH is a teaching hospital with 800 beds. As a specialized hospital for infectious diseases, we have a separate ED for patients with infectious diseases, and a large proportion of patients come to this ED for RTIs in winter. Participants were enrolled from the ED in BJDH, including patients with a fever or feeling feverish, or experiencing a cough, runny nose or stuffy nose, and fatigue or tiredness, who could be recruited directly from their first arrival at the ED, and possibly also presenting with vomiting or diarrhea. Patients were excluded if patients had experienced a fever for longer than 7 days or prescribed with antibiotics during the last 2 weeks, and we also excluded patients who presented in a state of unconsciousness, as well as those known to be HIV-infected. Written informed consent was obtained by the emergency department staff from the participants, or for children from the child's parents or guardians. This study was approved by the Ethics Committee of BJDH.

### 2.2. Clinical Staff Training

The FilmArray RP was the first multiplex test implemented in the ED by clinical staff, so all clinical staff were educated about the manufacturer's instructions by microbiology staff through personnel training on the operation of the FilmArray instrument. Clinical staff collected all the respiratory samples, consulting the standard operating procedure of clinical sample collection used in our hospital. Before the October start date of the study, all clinical staff undertook competency evaluations.

### 2.3. Sample Collection and FilmArray RP Assay

For the FilmArray RP assay, a nasopharyngeal swab for the respiratory sample was collected using a nylon flocked swab (Copan Diagnostics, Italy) at midturbinate, which was then placed in a viral transport medium (VTM, Copan Diagnostics) immediately. If respiratory virus detection was needed, the clinicians would take the nasopharyngeal swab from the patient' nasopharynx for more than 5 seconds directly, and all results from the FilmArray RP assay were recorded in the statistics records. The clinicians performed the FilmArray could then give an antibiotic prescription according to the patient's clinical manifestation, based on the results of the laboratory examination and the FilmArray RP assay. A trained assistant collected all clinical data and etiological information using a standard case report form and input this information into an electronic study database.

In this study, the FilmArray assay (FilmArray RP panel, BioFire Diagnostics, LLC, Salt Lake City, Utah) detected 20 targets of viral and atypical pathogens present in respiratory samples, including 17 respiratory viruses from the rhinovirus/enterovirus family (Rhino/Entero), respiratory syncytial virus (RSV), adenovirus (AdV), human metapneumovirus (hMPV), influenza B virus (Flu B), influenza A virus unsubtyped/H1/H3/2009H1 (Flu A/unsubtyped, Flu A/H1, Flu A/H3, and Flu A/2009H1), coronavirus 229E/HKU1/NL63/OC43 (Cov 229E, Cov HKU1, Cov NL63, and Cov OC43), parainfluenza 1/2/3/4 (PIV 1, PIV 2, PIV 3, and PIV 4), the bacterium *Bordetella pertussis* (*B*. *pertussis*), and two atypical pathogens including *Chlamydia pneumoniae* (*C*. *pneumoniae*) and *Mycoplasma pneumoniae* (*M*. *pneumoniae*) [20]. Rehydration buffer (1.0 ml) and the VTM (200 *μ*l) were loaded into the testing pouch by clinical staff under a ventilation hood. After scanning the sample identity document, automatic nucleic acid extraction, RNA reverse transcription, and the first-stage and second-stage multiplex PCR amplification were started successively, and the results were available in 65 min. For each target, the second-stage PCR amplification was undertaken in triplicate wells. The FilmArray assay automatically created the melting curve of the second-stage PCR to provide a positive or negative result. If internal control amplifications reported a negative result, the assay was defined as “Invalid,” with no pathogens.

### 2.4. Statistical Analysis

Patient demographic characteristics were collected, including age and gender. The clinical syndrome information included fever, cough, duration of symptoms, and if complicated with pneumonia. The clinical outcomes evaluated were antibiotic prescription and anti-influenza prescription. The data management software was Microsoft Excel 2015. All analyses in this study used IBM SPSS Statistics software v22.0 (StataCorp; USA) or Prism v6.0 (GraphPad Software; USA). The categorical variables of demographic and clinical characteristics and the clinical outcomes were shown in percentages and compared with Pearson's chi-square test or Fisher's exact test for differences. The white cell count, level of C-reactive protein, and lymphocytes were presented as a median (interquartile range). The differences in antimicrobials among groups with different pathogens detected were compared using Student's *t*-test or Mann–Whitney *U* test. *P* values of <0.05 were considered statistically significant.

## 3. Results

### 3.1. Demographic and Clinical Characteristics

**Figure 1:**
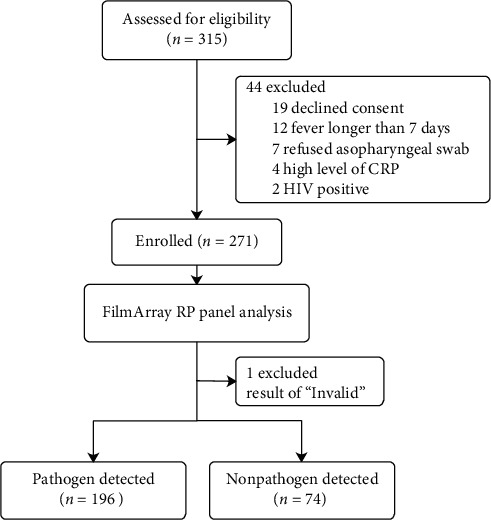
Flowchart of study participants. The results from the detection of respiratory pathogens using the FilmArray respiratory panel.

Between 15 October 2016 and 31 February 2017, the study assessed 315 patients for eligibility, of whom 271 were eligible to participate. None of the participants withdrew from the study (Figure 1). Forty-four (24.0%) of the patients were excluded as 19 declined to give consent, 12 had fever that lasted for longer than 7 days, 7 patients refused the nasopharyngeal swab, 4 had a high level of CRP (>50 mg/L), and HIV was positive for 2 patients. Grouped by the results of the FilmArray RP assay, the characteristics of the pathogen detected group and nonpathogen detected group are shown in Table 1. One hundred and ninety-six (72.6%) patients were detected as having one or more pathogens. A total of 93 (47.4%) patients were <16 years, but those aged between 16 and 49 accounted for the larger proportion in the nonpathogen detected group. There were high percentages of the clinical characteristics of fever (81.1%) and coughing (83.8%) in the nonpathogen detected group versus the pathogen detected group (60.7% and 61.7%, respectively). The median levels for the white cell count and C-reactive protein were higher in the nonpathogen detected group compared to the pathogen detected group (nonpathogen detected group 9.28 × 10^9^/L (IQR: 4.05–11.86) and 28.86 mg/L (IQR: 7.40–34.83); pathogen detected group 8.5 × 10^9^/L (IQR: 4.52–9.40), 16.70 mg/L (IQR: 2.40–19.30)).

### 3.2. Analysis of Signal Pathogens

Overall, 151 (55.9%) of the 270 patients had a signal pathogen detected (Table 2). Patients with a signal pathogen detected were divided into three groups according to age; children aged ≤16 years (*n* = 114) were the largest group in our study. The number of people in the age groups 16–49 and ≥50 years was small compared with the youngest group, with 111 and 45 patients in each group, respectively.

The highest prevalence of targeted pathogens in the three age groups was Flu A: the prevalence of Flu A in the groups ≤16 years, 16–49 years, and ≥50 years was 22.8%, 32.4%, and 31.1%, respectively. More specifically, the highest rate of Flu A from three distinguishable subtypes was FluA/H3 in all three age groups, with a rate of 26.7% in the group ≥50 years, followed by 19.8% in the group aged 16–49 years and 14.0% in the group ≤16 years. The other respiratory pathogens, such as Rhino/Entero (6.3%), RSV (6.3%), Flu B (2.2%), Cov (1.5%), *M*. *pneumoniae* (4.1%), and *B*. *pertussis* (3.3%) had low rates of less than 10.0%, and the detected rates of hMPV (0.7%), AdV (0.7%), and PIV (0.4%) were less than 1.0%. There was no detected *C*. *pneumoniae* at all in any of the 151 patients. As a common respiratory pathogen of pertussis in children, all nine cases of *B*. *pertussis* were detected only in the group ≤16 years. More detailed information is shown in Table 2.

### 3.3. Analysis of Copathogens

**Figure 2:**
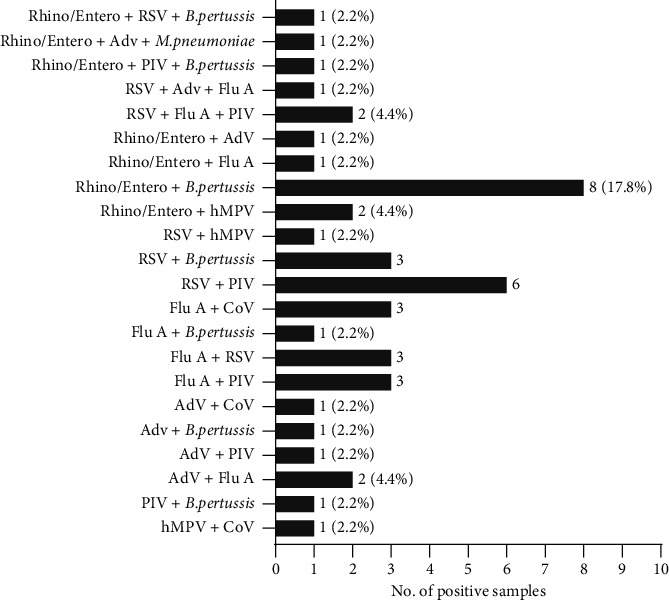
Prevalence of respiratory pathogens in the copathogens detected groups.

Among 270 patients, 45 had two or more respiratory pathogens codetected, with a rate of 16.7%. Among the 45 patients with codetected respiratory pathogens, 40 were codetected with dual pathogens, accounting for the majority of the sample. Four patients had triple pathogens codetected, and one had quadruple pathogens codetected. Rhino/Entero codetected with *B*. *pertussis* was the most common at 8 patients (17.8%), followed by 6 (13.3%) cases of RSV codetected with PIV. More detailed information is shown in Figure 2.

In the 45 cases with codetected respiratory pathogens, the total number of detected pathogens was 98; RSV and *B*. *pertussis* had the highest rate of 17.3%, followed by Flu A (16.3%), Rhino/Entero (15.3%), and PIV (15.3%). The rates of detection of AdV, hMPV, Cov, and *M*. *pneumoniae* were less than 10.0% (8.2%, 4.1%, 5.1%, and 1.0%, respectively). *C*. *pneumoniae* was not detected in any patients.

### 3.4. Outcome of Antimicrobial Prescriptions

According to the results of the FilmArray RP panel, 98, 98, and 74 patients were assigned to the Flu A/B virus detected group, non-Flu A/B virus pathogen detected group, and nonpathogen detected group, respectively. Antibiotic prescription rates were significantly different among the three groups (*χ*^*2*^ = 37.1, *P* < 0.001), with the highest rate of 50.0% occurring in the nonpathogen detected patients (Table 3). Between patients with a non-Flu A/B virus pathogen detected and those in the nonpathogen detected group, there was a significant difference in antibiotic prescription rates (28.8% and 51.4%, respectively ( *χ*^*2*^ = 8.2, *P* < 0.001)). The difference in anti-influenza prescription rates was also significant among the three groups (*χ*^*2*^ = 98.8, *P* < 0.001), with those with Flu A or Flu B receiving the most prescriptions (71.6%). Anti-influenza prescription rates were no different between patients with a non-Flu A/B virus pathogen detected and those in the nonpathogen detected group (3.1% and 8.1%, respectively (*χ*^*2*^ = 1.8, *P*=0.194)). More detailed information is shown in Table 3.

## 4. Discussion

This pragmatic clinical trial is the first, to our knowledge, to report on the performance of the FilmArray RP panel for direct use in an ED for outpatients by clinical staff, in terms of outcomes including the analysis of the signal pathogen detected, analysis of copathogens detected, and antimicrobial prescription rates. After clinical staff training and personnel training, 271 specimens were detected over the winter season using the automated nested multiplex PCR system for pathogen detection, yielding a detection rate of 72.6% and a positive result in 270 specimens. Although the PCR instrument was operated by clinical staff, only one sample failed. When the three groups were divided by age in the analysis of signal pathogen detection, the group ≤16 years had the highest positive rate of 81.6%, followed by the group ≥50 years (66.7%), and the group aged 16–49 years had the lowest positive rate of 65.8%. The trend of low detection rates of respiratory viral pathogens in adults is consistent with previous studies; Christine et al. showed that with increasing age, the positive rates obtained with the FilmArray RP panel decrease, and other studies using multiplex respiratory pathogen PCR have also reported lower detection rates in adults [3, 21, 22]. However, the overall detection rate in this study, which resulted from clinicians operations, was higher than that found in other studies of diagnosis with the FilmArray RP at tertiary care medical centers (aged 5 days to 91 years), EDs, in the urgent care of pediatric patients, or in ward-based operations with inpatient and outpatient medical areas [7, 15, 22, 23]. It is possible that ED-based POC testing takes less time from sample collection to performing a FilmArray run than testing in specific test settings. This finding may also relate to the differences in time and sample size, or the characteristics of the age distribution of the participants.

We also note here differences in the detected number of respiratory pathogens in results where a signal pathogen was detected. Flu A was the most detected viral pathogen in all three groups; Liu et al., Qian et al., and Jin et al. similarly observed that Flu A was the most detected pathogen in RTIs in winter in their studies [16, 24, 25]. Rhino/Entero and RSV were the predominant viruses for all age groups, but far less were detected than the most detected Flu A [19, 26]. Rhinovirus is a common virus in all seasons; however, peaks of rhinovirus infections are well documented around September, and a low positive rate of rhinovirus may be associated with that reason [3, 21].

*M*. *pneumoniae* was observed in all three groups, and the positive rates for the child group and the older adult group were higher than that for the group aged 16–49 years. In China, *M*. *pneumoniae* is reported as one of the most common pathogens of patients with CAP, and it was an unexpected result in our study that few cases were found [24, 27] with an overall detection rate of 4.1% among the 270 participants, further revealing the diagnostic performance of the FilmArray RP panel in ED applications. *B*. *pertussis* cases were detected only in the group ≤16 years with a positive rate of 7.9% in 114 patients; at present, the use of culture and serology methods in the diagnosis of pertussis is the only choice in China. The FilmArray RP panel may therefore help to manage patients with suspected pertussis, since it is difficult to identify other respiratory pathogens which often cause similar clinical symptoms to pertussis [28]. No *C*. *pneumoniae* cases were detected in our study; Beijing has a lowest incidence of reported *C*. *pneumonia* in China, with an incidence of 0.40% and 2.97% found by Chen et al. [29] and Zhao et al. [30], respectively.

More than one respiratory pathogen was codetected in 16.7% of the 270 specimens in this study. Li et al. reported a rate of 25.5% codetected patients from among children aged 19 days to 15 years with RTIs when using the FilmArray Respiratory Panel [16]. However, in adults, studies have mostly reported lower rates of approximately 10% to 15.9% [19, 21]. In terms of codetected respiratory pathogens, the largest proportion includes RSV (17.3%) and *B*. *pertussis* (17.3%), while Flu A was the second most common pathogen with a lower positive rate (16.3%) than in the analysis of signal detected pathogens. Because there was no laboratory testing for respiratory viral pathogens (except for Flu A and Flu B) at our hospital, the detection of multiplex respiratory organisms may be the most unexpected result for the clinical staff in our ED, fully illustrating the value of the FilmArray RP panel as a tool for POC testing in clinical departments.

We found that patients detected with Flu A and Flu B received significantly lower antibiotic prescriptions and more anti-influenza prescriptions than those who had a positive result for non-Flu A/B viral pathogens or had a negative result for all pathogens. The detection of a positive result for viral pathogens contributed to a decrease in the usage of antibiotics in patients with RTIs in ED, which suggested that the use of the FilmArray RP panel may be helpful in reducing antibiotic prescriptions in outpatients with RTIs. However, there was no evidence for a significant decrease in anti-influenza prescription usage between non-Flu A/B viral pathogen detected patients and nonpathogen detected patients. For those without influenza A or B virus identified, the clinicians were more likely to prescribe anti-influenza drugs for outpatients in the winter season, suggesting the prevention of inappropriate prescriptions of anti-influenza medications needs further attention [31]. As for the effects of respiratory viral pathogen results on antimicrobial prescription rates, including antibiotics and anti-influenzas, mixed findings have been reported by previous studies. In our study, the multiplex pathogen PCR system of the FilmArray showed the potential ability to reduce unnecessary antimicrobial prescriptions.

According to the results of the FilmArray RP panel, 98, 98, and 74 patients were assigned to the Flu A/B virus detected group, non-Flu A/B virus pathogen detected group, and nonpathogen detected group, respectively. Antibiotic prescription rates were significantly different among the three groups (*χ*^*2*^ = 37.1, *P* < 0.001), with the highest rate of 50.0% occurring in nonpathogen detected patients (Table 3). Between patients with a non-Flu A/B virus pathogen detected and those who were in the nonpathogen detected group, there was a significant difference in antibiotic prescription rates (28.8% and 51.4%, respectively (*χ*^*2*^ = 8.2, *P* < 0.001)). The differences in anti-influenza prescription rates were also significant among the three groups (*χ*^*2*^ = 98.8, *P* < 0.001), with those with Flu A or Flu B receiving the most prescriptions (71.6%). Anti-influenza prescription rates were no different between patients with a non-Flu A/B virus pathogen detected and those in the nonpathogen detected group (3.1% and 8.1%, respectively, ( *χ*^*2*^ = 1.8, *P*=0.194)). More detailed information is shown in Table 3.

However, there are a number of limitations in this study. First, our study was a single-center study, and results from multicenter need to be reported for verifying. Second, another possible limitation of our study is the bias from age among participants, as we all know, young children in which infected with RTI have a totally different percentages of respiratory pathogenic spectra. Third, because of the high cost of the FilmArray RP panel, we choose to implement our study only in a single winter season and we do not have access data covering a complete year. Fourth, the study was conducted in an ED which is specialized for infectious diseases, rather than general ED that included clinical staff facing a range of diseases. Finally, the utility of the FilmArray respiratory panel in the emergency department needs further evaluation in multicenter studies and with more patients in EDs.

## 5. Conclusion

We tested respiratory pathogens in patients with RTIs in an ED using the FilmArray respiratory panel undertaken by clinical staff, for the first time in China. We found that the FilmArray, an automated nested multiplex diagnostic PCR system, was a rapid and simple tool for clinical staff to utilize in a nonspecific test setting. The multiplex diagnostic system significantly increased the rate of respiratory pathogen detection, and clinical staff from the ED performed the FilmArray RP testing without too many “Invalid” results, suggesting that this diagnostic system has potential for use in many divergent clinical settings. Based on the results of the FilmArray RP panel, the unnecessary usage of antibiotic prescriptions and anti-influenza prescriptions in patients with nondetected pathogens may be reduced when they present to an ED.

## Figures and Tables

**Table 1:** General characteristics of enrolled patients.

	Pathogen detected	Nonpathogen detected
Male gender	53.1% (104/196)	55.4% (41/74)
No. (%) of patients of age		
≤16 years	93 (47.4)	21 (28.4)
16–49 years	73 (37.2)	38 (51.4)
≥50 years	30 (15.4)	15 (20.4)
No. (%) of patients of prescription during the last 2 weeks		
Antibiotic used	10 (5.1)	6 (8.1)
Anti-influenza used	6 (3.1)	7 (10.8)
No. (%) of patients of fever	119 (60.7)	60 (81.1)
No. (%) of patients of coughing	121 (61.7)	62 (83.8)
Duration of symptoms (days)^a^	2.3 (1–2)	3.4 (2–3)
Complicated with pneumonia (%)	40 (20.4)	24 (32.4)
White cell count (×109/L)^a^	8.5 (4.52–9.40)	9.28 (4.05–11.86)
C-reactive protein (mg/L)^a^	16.70 (2.40–19.30)	28.86 (7.40–34.83)
Lymphocytes (×109/L)^a^	4.64 (1.26–5.73)	3.03 (0.91–1.75)

^a^Median (IQR). Duration of symptoms: the time from patients had symptoms to visit the emergency department.

**Table 2:** Prevalence of respiratory pathogens signal detected in different age groups.

Result	≤16 years		16–49 years		≥50 years		Total	
	No.	Prevalence (%, *n* = 114)	No.	Prevalence (%, *n* = 111)	No.	Prevalence (%, *n* = 45)	No.	Prevalence (%, *n* = 270)
Not detected	21	18.4	38	34.2	15	33.3	74	27.4
Rhino/Entero	6	5.3	7	6.3	4	8.9	17	6.3
RSV	8	7.0	7	6.3	2	4.4	17	6.3
AdV	1	0.9	1	0.9	0	0.0	2	0.7
hMPV	0	0.0	2	1.8	0	0.0	2	0.7
Flu B	2	1.8	2	1.8	2	4.4	6	2.2
FluA/H1	0	0.0	0	0.0	0	0.0	0	0.0
FluA/H3	16	14.0	22	19.8	12	26.7	50	18.5
FluA/2009 H1	8	7.0	7	6.3	0	0.0	15	5.6
Flu A unsubtyped	2	1.8	7	6.3	2	4.4	11	4.1
Flu A total	26	22.8	36	32.4	14	31.1	76	28.1
Cov 229E	0	0.0	1	0.9	2	4.4	3	1.1
Cov HKU1	0	0.0	1	0.9	0	0.0	1	0.4
Cov NL63	0	0.0	0	0.0	0	0.0	0	0.0
Cov OC43	0	0.0	0	0.0	0	0.0	0	0.0
Cov total	0	0.0	2	1.8	2	4.4	4	1.5
PIV1	2	1.8	0	0.0	0	0.0	2	0.7
PIV2	0	0.0	0	0.0	0	0.0	0	0.0
PIV3	3	2.6	0	0.0	1	2.2	4	1.5
PIV4	0	0.0	1	0.9	0	0.0	1	0.4
PIV total	5	4.4	1	0.9	1	2.2	7	2.6
*C*. *pneumoniae*	0	0.0	0	0.0	0	0.0	0	0.0
*M*. *pneumoniae*	6	5.3	3	2.7	2	4.4	11	4.1
*B*. *pertussis*	9	7.9	0	0.0	0	0.0	9	3.3

Rhino/Entero: rhinovirus/enterovirus; RSV*:* respiratory syncytial virus; AdV adenovirus; hMPV: human metapneumovirus; Flu B: influenza B virus; Flu A/unsubtyped, FluA/H1, FluA/H3, FluA/2009H1: influenza A virus unsubtyped/H1/H3/2009H1; Cov 229E, Cov HKU1, Cov NL63, Cov OC43: coronavirus 229E/HKU1/NL63/OC43; PIV 1/2/3/4: parainfluenza 1/2/3/4; *B*. *pertussis*: *Bordetella pertussis*; *C*. *pneumoniae*: *Chlamydia pneumoniae*; *M*. *pneumoniae*: *Mycoplasma pneumoniae*.

**Table 3:** Antimicrobial prescription rates among patients.

	No. (%) of patients with			Total	*χ* ^*2*^	*P* value
	Flu A/B	Non-Flu A/B viral pathogen	Nonpathogen			
Total patients included	97	52	74	223		
Antibiotic prescription	9 (9.3)	15 (28.8)	38 (51.4)	62 (27.8)	37.1	＜0.001
Oseltamivir prescription	70 (72.2)	6 (11.5)	4 (5.4)	80 (35.9)	98.8	＜0.001

## Data Availability

All major data generated or analyzed during this study were included in this article.

## Abbreviations

FilmArray RP: FilmArray respiratory panel
RTI: Respiratory tract infections
ED: Emergency department
CAP: Community-acquired pneumonia
DFA: Direct fluorescence assays
TAT: Turnaround time
POC: Point of care
BJDH: Beijing Ditan Hospital
CRP: C-reactive protein
HIV: Human immunodeficiency virus
VTM: Viral transport media.

## References

[1] Brendish N. J., Malachira A. K., Armstrong L. (2017). Routine molecular point-of-care testing for respiratory viruses in adults presenting to hospital with acute respiratory illness (ResPOC): a pragmatic, open-label, randomised controlled trial. *The Lancet Respiratory Medicine*.

[2] McAllister D. A., Liu L., Shi T. (2019). Global, regional, and national estimates of pneumonia morbidity and mortality in children younger than 5 years between 2000 and 2015: a systematic analysis. *The Lancet Global Health*.

[3] Self W. H., Williams D. J., Zhu Y. (2016). Respiratory viral detection in children and adults: comparing asymptomatic controls and patients with community-acquired pneumonia. *Journal of Infectious Diseases*.

[4] Childs A., Zullo A. R., Joyce N. R. (2019). The burden of respiratory infections among older adults in long-term care: a systematic review. *BMC Geriatrics*.

[5] Ma X., Conrad T., Alchikh M., Reiche J., Schweiger B., Rath B. (2018). Can we distinguish respiratory viral infections based on clinical features? A prospective pediatric cohort compared to systematic literature review. *Reviews in Medical Virology*.

[6] Wabe N., Li L., Dahm M. R. (2019). Timing of respiratory virus molecular testing in emergency departments and its association with patient care outcomes: a retrospective observational study across six Australian hospitals. *BMJ Open*.

[7] Rappo U., Schuetz A. N., Jenkins S. G. (2016). Impact of early detection of respiratory viruses by multiplex PCR assay on clinical outcomes in adult patients. *Journal of Clinical Microbiology*.

[8] Nair H., Simões E. A., Rudan I. (2013). Global and regional burden of hospital admissions for severe acute lower respiratory infections in young children in 2010: a systematic analysis. *The Lancet*.

[9] Pneumonia Etiology Research for Child Health (PERCH) Study Group (2019). Causes of severe pneumonia requiring hospital admission in children without HIV infection from Africa and Asia: the PERCH multi-country case-control study. *Lancet*.

[10] Tatarelli P., Magnasco L., Borghesi M. L. (2019). Prevalence and clinical impact of VIral Respiratory tract infections in patients hospitalized for Community-Acquired Pneumonia: the VIRCAP study. *Internal and Emergency Medicine*.

[11] Crotty M. P., Meyers S., Hampton N. (2015). Impact of antibacterials on subsequent resistance and clinical outcomes in adult patients with viral pneumonia: an opportunity for stewardship. *Critical Care*.

[12] Ding G., Vinturache A., Lu M. (2019). Addressing inappropriate antibiotic prescribing in China. *Canadian Medical Association Journal*.

[13] Mahony J. B., Petrich A., Smieja M. (2011). Molecular diagnosis of respiratory virus infections. *Critical Reviews in Clinical Laboratory Sciences*.

[14] Duan S., Gu X., Fan G. (2019). Evaluation of a molecular point-of-care testing for viral and atypical pathogens on intravenous antibiotic duration in hospitalized adults with lower respiratory tract infection: a randomized clinical trial. *Clinical Microbiology and Infection*.

[15] Busson L., Bartiaux M., Brahim S. (2019). Contribution of the FilmArray respiratory panel in the management of adult and pediatric patients attending the emergency room during 2015-2016 influenza epidemics: an interventional study. *International Journal of Infectious Diseases*.

[16] Li J., Tao Y., Tang M. (2018). Rapid detection of respiratory organisms with the FilmArray respiratory panel in a large children’s hospital in China. *BMC Infectious Diseases*.

[17] Brendish N. J., Malachira A. K., Clark T. W. (2017). Molecular point-of-care testing for respiratory viruses versus routine clinical care in adults with acute respiratory illness presenting to secondary care: a pragmatic randomised controlled trial protocol (ResPOC). *BMC Infectious Diseases*.

[18] Xu M., Qin X., Astion M. L. (2013). Implementation of filmarray respiratory viral panel in a core laboratory improves testing turnaround time and patient care. *American Journal of Clinical Pathology*.

[19] Andrews D., Chetty Y., Cooper B. S. (2017). Multiplex PCR point of care testing versus routine, laboratory-based testing in the treatment of adults with respiratory tract infections: a quasi-randomised study assessing impact on length of stay and antimicrobial use. *BMC Infectious Diseases*.

[20] Poritz M. A., Blaschke A. J., Byington C. L. (2011). FilmArray, an automated nested multiplex PCR system for multi-pathogen detection: development and application to respiratory tract infection. *PLoS One*.

[21] Litwin C. M., Bosley J. G. (2014). Seasonality and prevalence of respiratory pathogens detected by multiplex PCR at a tertiary care medical center. *Archives of Virology*.

[22] Leber A. L., Everhart K., Daly J. A. (2018). Multicenter evaluation of BioFire FilmArray respiratory panel 2 for detection of viruses and bacteria in nasopharyngeal swab samples. *Journal of Clinical Microbiology*.

[23] Lee S. H., Ruan S. Y., Pan S. C., Lee T. F., Chien J. Y., Hsueh P. R. (2019). Performance of a multiplex PCR pneumonia panel for the identification of respiratory pathogens and the main determinants of resistance from the lower respiratory tract specimens of adult patients in intensive care units. *Journal of Microbiology, Immunology and Infection*.

[24] Liu Y. F., Gao Y., Chen M. F., Cao B., Yang X. H., Wei L. (2013). Etiological analysis and predictive diagnostic model building of community-acquired pneumonia in adult outpatients in Beijing, China. *BMC Infectious Diseases*.

[25] Qian Y., Ai J., Wu J. (2019). Rapid detection of respiratory organisms with FilmArray respiratory panel and its impact on clinical decisions in Shanghai, China, 2016–2018. *Influenza and Other Respiratory Viruses*.

[26] Chen J., Li X., Wang W., Jia Y., Lin F., Xu J. (2019). The prevalence of respiratory pathogens in adults with community-acquired pneumonia in an outpatient cohort. *Infection and Drug Resistance*.

[27] Qu J., Yang C., Bao F., Chen S., Gu L., Cao B. (2018). Epidemiological characterization of respiratory tract infections caused by Mycoplasma pneumoniae during epidemic and post-epidemic periods in North China, from 2011 to 2016. *BMC Infectious Diseases*.

[28] Xu Y. H., Wang L., Xu J. (2014). Seroprevalence of pertussis in China. *Human Vaccines & Immunotherapeutics*.

[29] Chen L., Zhou F., Li H. (2018). Disease characteristics and management of hospitalised adolescents and adults with community-acquired pneumonia in China: a retrospective multicentre survey. *BMJ Open*.

[30] Zhao M.-C., Li G.-X., Zhang D. (2017). Clinical evaluation of a new single-tube multiplex reverse transcription PCR assay for simultaneous detection of 11 respiratory viruses, Mycoplasma pneumoniae and Chlamydia in hospitalized children with acute respiratory infections. *Diagnostic Microbiology and Infectious Disease*.

[31] Echavarría M., Marcone D. N., Querci M. (2018). Clinical impact of rapid molecular detection of respiratory pathogens in patients with acute respiratory infection. *Journal of Clinical Virology*.

